# Mandibular Third Molar Impaction among Patients Visiting Outpatient Dental Department of a Tertiary Care Centre

**DOI:** 10.31729/jnma.8295

**Published:** 2023-10-31

**Authors:** Narayan Sharma Lamichhane, Bhawana Sigdel, Sushma Lamichhane, Rupam Tripathi, Ujjwal Koirala, Dwarika Prasad Bajgai

**Affiliations:** 1 Department of Dental Surgery, Pokhara Academy of Health Sciences, Western Regional Hospital, Pokhara, Kaski, Nepal; 2 Department of Oral and Maxillofacial Surgery, Gandaki Medical College, Pokhara, Kaski, Nepal; 3 Department of Oral and Maxillofacial Surgery, Nepalgunj Medical College Teaching Hospital, Nepalgunj, Banke, Nepal

**Keywords:** *impacted tooth*, *pericoronitis*, *prevalence*, *third molar*

## Abstract

**Introduction:**

Mandibular third molar impaction is the most common impaction as third molars are last to erupt at the age of 17-25 years. Despite its high prevalence and negative impacts, there is limited study about mandibular third molar impaction. The aim of this study is to find out the prevalence of mandibular third molar impaction among patients visiting the outpatient Dental Department of a tertiary care centre.

**Methods:**

A descriptive cross-sectional study was conducted among patients visiting the Outpatient Dental Department of a tertiary care centre from 1 January 2023 to 30 June 2023. Ethical approval was taken from the Institutional Review Committee of the same institute. A total of 414 orthopantomograms were prospectively reviewed for the angulation of impaction, depth and position. The indication for extraction was recorded from patients' cards during the time of extraction. Convenience sampling method was used. The point estimate at a 95% confidence interval was calculated.

**Results:**

The prevalence of mandibular third molar impaction was 37.13% (34.29-39.97, 95% Confidence Interval). There was a high prevalence of mesioangular 344 (45.38%) pattern followed by vertical 249 (32.85%) for mandibular third molar impaction, the most commonly involved group was 20-30 years, with female 247 (59.70%) predominance. Bilateral impaction 344 (83.10%) was more prevalent than unilateral. Depth A, 639 (84.3%), ramus relation level I, 602 (79.42%) was the most common level of impaction. Recurrent pericoronitis 160 (38.6%) was the common indication for extraction followed by dental caries 145 (35%).

**Conclusions:**

The prevalence of mandibular third molar impaction was lower than other similar studies done in similar settings.

## INTRODUCTION

An Impacted tooth or an embedded tooth is a tooth that has failed to erupt to its correct position in the dental arch and its eruption potential has been lost. Impaction is defined as a cessation of the eruption of a tooth caused by a clinically or radiographically detectable physical barrier in the eruption path or by an ectopic position of the tooth.^1^ The mandibular third molar is the most commonly impacted tooth in the oral cavity accounting for 98% of all impacted teeth.^2^

Impacted teeth are associated with numerous disorders in the orofacial regions such as caries, pericoronitis, cystic lesions, periodontitis, neoplasms, or root resorption.^3^ There are very few studies done to date.

The aim of this study is to find out the prevalence of mandibular third molar impaction among patients visiting the outpatient Dental Department of a tertiary care centre.

## METHODS

This descriptive cross-sectional study was conducted in the Dental Department, Pokhara Academy of Health Sciences, Western Regional Hospital, Pokhara, Nepal. The patients visiting the department from 1 January 2023 to 30 June 2023 fulfilling the eligibility criteria were included in this study. Permission for study and data collection was taken from the Institutional Review Committee (Reference number: 121/079). Informed consent was obtained from the patient for the use of radiographs and clinical records. Convenience sampling method was used. The data was collected from clinical records for indication of extraction and orthopantomograms (OPGs) for the presence, site, angulation, depth and ramus relationship of impaction of the third molar. Inclusion criteria include a patient with a complete clinical record, a minimum age group of 20 years, good quality panoramic radiographs, a patient with no history of any lower third molar extraction, the presence of a second molar adjacent to the impacted third molar, a patient with no filling for wisdom teeth or the second molar. Exclusion criteria include patients below 20 years of age, and incomplete clinical radiological records, severe systemic disease conditions, craniofacial anomalies or syndromes such as achondroplasia, progeria, oxycephaly, cleidocranial dysostosis, and Down's syndrome, any previous trauma or pathology. The sample size was calculated by using the following formula:


$$\begin{array}{ll} \text{n} & ={\text{Z}}^{2}\times \frac{\text{p}\times \text{q}}{{\text{e}}^{\text{2}}}\\ & =1.96^{2}\times \frac{0.50\times 0.50}{0.03^{2}}\\ & =1068 \end{array}$$

Where,

n = minimum required sample size
Z = 1.96 at 95% Confidence Interval
p = prevalence taken as 50% for maximum sample size calculation
q = 1-p
e = margin of error, 3%

The total calculated sample size was 1068. However, 1115 sample size was taken.

The angulation of impacted lower third molars was measured using Winter's classification^4^ and recorded as vertical, mesioangular, horizontal and distoangular. The classification of impaction was measured using Quek, et al. (2003) classification,^5^ as follows

(i) Vertical impaction: 10 to -10°
(ii) Mesioangular impaction: 11 to 79°
(iii) Horizontal impaction: 80 to 100°
(iv) Distoangular impaction: -11 to -79°
(v) Other (inverted, buccolingual): 111 to -80°

Uncommon angulations (mesioinverted, distoinverted, and distohorizontal) were categorized under others.

The level of impaction of the mandibular third molar was recorded using Pell and Gregory classification^6^ as level A, level B, and level C. Similarly, the relationship of the impacted third molar with the ramus of the mandible and second molar was also recorded using Pell and Gregory classification^6^ as Class I, Class II and Class III.

The indications for the removal of the third molar were recorded from patients' clinical records which included pericoronitis, caries, periodontitis, both pericoronitis and caries, pre-prosthetic preparation, prophylactic removal, cyst tumour etc. All OPGs were taken using SIDEXIS XG software, version 2005 (Sirona Dental System GmbH, Bensheim, Germany). To avoid any discrepancy in judgment, all the OPGs were reviewed meticulously by a single examiner with the help of a well-illuminated X-ray viewer.

Data was analysed using IBM SPSS Statistics version 25.0. The point estimate at a 95% confidence interval was calculated.

## RESULTS

Among 1115 patients, mandibular third molar impaction was found in 414 (37.13%) (34.29-39.97, 95% CI). A total of 344 (83.09%) patients had bilateral impaction and 70 (16.90%) had unilateral impaction. The mean age of the patient was 37.90±13.85 years ranging from 20 to 73 years. There were 247 (59.7%) females and 167 (40.30%) males with a female-to-male ratio of 1.47 (Table 1).

**Table 1:** Sexwise distribution of patients with mandibular third molar impaction (n= 414).

Gender	Bilateral n (%)	Unilateral n (%)
Male	137 (33.09)	30 (7.24)
Female	207 (50)	40 (9.66)

The cases were divided into 4 age groups and a higher prevalence of bilateral impaction was seen in the 20-30 years age group (Table 2).

**Table 2:** Agewise distribution (n= 414).

Age-group (years)	Bilateral n (%)	Unilateral n (%)
20-30	139 (33.57)	12 (2.89)
31-40	100 (24.15)	12 (2.89)
41-50	49 (11.83)	17 (4.10)
>50	56 (13.53)	29 (7)

The proportion of impacted mandibular third molar was equal between the right and left sides in males and higher on the left side than the right side in females (Table 3).

**Table 3:** Unilateral distribution based on sex (n= 70).

Gender	Left n (%)	Right n (%)
Male	15 (21.43)	15 (21.43)
Female	24 (34.28)	16 (22.86)

Mesioangular type of impaction was the most common impaction followed by vertical, horizontal and distoangular respectively. Regarding depth and position of impaction, Level B impaction was seen in the majority and class-I was the predominant finding (Table 4).

**Table 4:** Angulation, Depth, and Ramus of impacted mandibular third molar (n= 758).

Angulation	38 (Left mandibular 3rd molar) n (%)	48 (Right mandibular 3rd molar) n (%)
Mesioangular	176 (46.10)	168 (44.70)
Horizontal	68 (17.80)	58 (15.40)
Vertical	120 (31.4)	129 (34.30)
Distoangular	18 (4.70)	21 (5.60)
**Depth**	**38 (Left mandibular 3rd molar)**	**48 (Right mandibular 3rd molar)**
Depth A	323 (84.55)	316 (84.05)
Depth B	33 (8.64)	36 (9.57)
Depth C	26 (6.81)	24 (6.38)
**Ramus Relationship**	**38 (Left mandibular 3rd molar)**	**48 (Right mandibular 3rd molar)**
Level I	308 (80.63)	294 (78.2)
Level II	45 (11.78)	59 (15.70)
Level III	29 (7.59)	23 (6.10)

Pericoronitiswas the mostcommon indicationfor removal of third molar followed by caries and periodontitis (Table 5).

**Table 5:** Indications for extraction of impacted mandibular third molar (n = 414).

Indications	n (%)
Pericoronitis	160 (38.64)
Dental Caries	145 (35.02)
Periodontitis	32 (7.73)
Prophylactic Removal	26 (6.28)
Facial pain	24 (5.79)
Dental caries on 2nd molar	21 (5.07)
Prosthodontic reason	6 (1.45)

## DISCUSSION

The prevalence of mandibular third molar impaction was 37.13%, which is lower than other studies in Singapore Chinese, Iran, Indian and Turkey population and similar to studies in Nepalese population.^5,7-11^

In the present study, the prevalence among females was 59.66% and that of males was 40.33%. This finding is in agreement with previous studies,^1,5,8,10^ and is in contrast with other studies where male predominates.^12^ The tendency to impact the third molar in females may be caused by different growth patterns between males and females. Growth spurt in females occurs in 10-12 years and 12-14 years in males. The pattern of female skeletal growth is rapid and brief, while it's slow and long in males. Males continue to expand their jaw during the third molar's eruption whereas females stop growing their jaws when the third molars erupt leaving room for the emergence of the molars in males.^13^ Furthermore, the end of the growth spurt in females is related to their onset of menarche. This has arrived earlier by 2.8 years in the modern world owing to intrinsic factors like living conditions and lifestyle. This may offer a relationship of early physical maturity and late mineralization of third molars to the higher frequency in females.^14^

The prevalence of impaction in different populations ranges from 9.5 to 68% according to various authors.^5,7^ In our study impaction was higher in the 20-30 years age group which is in agreement with other studies.^1,11,13^ There exists a correlation between impaction and age. It is higher in the younger age group of 20-30 years and decreases as people mature. One of the studies in their 12-year observation of third molars in adults suggests that third molars undergo continuous clinical change at least up to the age of 32 years.^15^ We observed 36.46% impaction in the 20-30 age group, 27.04%, 15.93% and 20.53% in the 31-40, 41-50 and >51 years age group respectively. A slightly higher percentage in >51 years age group may be attributed to the wide range of age group 20-73 years included in the study.

The reason for the high prevalence of impacted mandibular third molars is related to a myriad of factors. The discrepancy in the prevalence of the impacted third molars may be due to genetic or racial differences.^8^ Furthermore, the jaw size in relation to the cumulative teeth size has been identified as a contributing factor, which may result from the difference in dietary habits.^16^ The transition from a rougher, fibrous diet low in sugar to a more refined, softer, western diet does not offer a dedicated effort in mastication, resulting in loss of growth stimulation of jaws. The refined diet of modern society requires less masticatory forces because of which interproximal wear or attrition is absent contributing to the impaction of third molar.

In our study, bilateral cases 83.09% were more common than unilateral 70 (16.90%). In unilateral cases; the left side was more affected than the right. The bilateral finding is consistent with other studies.^3,5^ The angulation pattern of the current study is similar to other studies conducted in Nepal where the common type of angulation was mesioangular and distoangular being the least.^10,11^ Wide variation has been seen regarding the pattern of mandibular impaction across the world. Studies conducted in the Indian population,^9^ Chinese population,^5^ Iranian population,^8^ and Saudi populations^16^ show mesioangular impaction dominance in contrast to studies conducted in the Jordanian,^12^ and Turkish^7^ populations that exhibit vertical impaction to be the most common. According to the review literature, root formation is the primary period during which the occlusal surface changes from a mesial-straight to a vertical-straight orientation. It is therefore possible that during this phase, the tooth rotates primarily from a horizontal to a mesioangular to a vertical position. Belfast Study group at Queen's University argued that depending on the level of development in the root, differences in mesial and distal roots' rates of root growth cause roots to either remain mesially or shift to a vertical position.^17^ The higher prevalence of mesioangular impaction may be related to the more common underdevelopment of mesial roots.

The level of impaction indicates the depth at which the tooth is buried in bone. The level of impaction for the mandibular third molar was higher in level A accounting for (84.30%) followed by level B, (9.10%) and level C, (6.60%). Our finding is in agreement with other studies,^9^ and in contrast with other studies having level B as the most common.^5^

The eruption of the third molar is usually foreseen based on the amount of space available between the second molar and the ascending ramus of the mandible. If the available mesiodistal space is equal to or greater than the mesiodistal width of the crown, then there is a 70% probability for its eruption.^9^ The most common ramus relationship is level I, (79.42%) which is consistent with other studies^1^ and contrasts other studies in which the classic II ramus relationship is most prevalent.^8,9^ The least occurrence is class III, (6.86%), which is in accordance with most of the other studies.^1^

Extraction of the third molar is the most frequently performed procedure in oral and maxillofacial surgery. The various indications for its removal could be because of pain, swelling, acute or chronic pericoronitis, dental caries on 3^rd^ molar, periodontitis, presence of cyst or tumour, food impaction, caries of second molar, pulpal involvement, facial pain, prosthetic rehabilitation, orthodontic consideration, prophylactic removal and preparation of orthognathic surgery.^10^

Recurrent pericoronitis was the most common indication for third molar extraction in our study which is supported by other studies^11,12^ and contrasts with the another study done in Nepal.^10^ where pain due to dental caries is the most common cause for extraction. Pericoronitis is an inflammation of soft tissue surrounding the crown of a partially erupted tooth, which is prevalent in young adults in the period between 16 and 30 years of age with the highest incidence in 21-25 year-old, which is also a time for third molar eruption.

Caries involvement was the second most common indication for removal which is in agreement with other similar studies.^11,12,6^ The depth of the impacted third molar and the occlusal angulation between the impacted tooth and the occlusal surface of the second molar influence the distal caries in the second molar.^18^ An impacted third molar makes the adjacent second molar susceptible to an array of pernicious effects such as caries, periodontitis, cervical resorption, and root resorption. Another similar study suggests prophylactic removal of third molars so as to avoid consequences of distal caries in the mandibular second molar.^18^ Periodontal pathology was the third most common cause of extraction in our study. Similar incidences of high periodontal pathology and dental caries on either 2^nd^ or 3^rd^ or both the molars were seen in the age group above 40 years in other studies too.^19^ There are many studies supporting an association between chronic facial pain and the presence of an impacted third molar.^12^ This is in consensus with the current study where chronic facial pain is one of the indications for third molar removal.

Guidelines for the management of impacted mandibular third molar proposed by the National Institute for Clinical Excellence (NICE) recommend against the prophylactic removal of the third molar which is pathologically free indicating no reliable research to suggests that this practice benefits patients and patients who do have healthy wisdom teeth removed are being exposed to the risks of surgery.^20^ However, in this study 24 cases, 6.3% underwent prophylactic removal, which is in contrast with the NICE guidelines.

The indication for removal was to salvage the second molar, the identical pattern of impaction where one of the sides was symptomatic and required removal, to avoid repeated episodes of pain and swelling of the functionless tooth on the contralateral side despite different angulation, and most commonly to avoid economic hassle as they were travelling abroad for further studies where surgical extraction was considered expensive and were suggested to do so by their friends and relatives. Similar indications were seen in other studies as well.^10^

Not all impacted third molar pose a risk to the health and well-being of an individual as some may remain asymptomatic throughout life. Approaches for asymptomatic pathology-free third molar range from watchful waiting until the problem manifests to routine prophylactic removal at an early age. Now the surgeon has a pivotal role in deciding whether to counsel the patient for extraction or not. But in a country like Nepal where regular follow-up for all is not feasible due to socioeconomic conditions, lack of proper dental services at the local level and geographical terrain, a judicious decision has to be made for third molar extraction.

Since the study is a single institution-based descriptive cross-sectional study with a limited sample size, the results might not be completely generalizable in other settings.

## CONCLUSIONS

The prevalence of mandibular third molar impaction was lower than other international studies and similar to studies in the Nepalese population conducted in similar settings. Surgeons should aware patients for potential complications and timely intervention. Multicentre, large sample size and randomized control trial would be better to substantiate the usefulness of the study.

## References

[1] Kumar VR, Yadav P, Kahsu E, Girkar F, Chakraborty R (2017). Prevalence and pattern of mandibular third molar impaction in Eritrean population: a retrospective study.. J Contemp Dent Pract..

[2] Badawi Fayad J, Levy JC, Yazbeck C, Cavezian R, Cabanis EA (2004). Eruption of third molars: relationship to inclination of adjacent molars..

[3] Zaman MU, Almutairi NS, Abdulrahman Alnashwan M, Albogami SM, Alkhammash NM, Alam MK (2021). Pattern of mandibular third molar impaction in nonsyndromic 17760 patients: a retrospective study among Saudi population in Central Region, Saudi Arabia.. Biomed Res Int..

[4] Winter GB (1926). Principles of exodontia as applied to the impacted mandibular third molar: a complete treatise on the operative technic with clinical diagnoses and radiographic interpretations..

[5] Quek SL, Tay CK, Tay KH, Toh SL, Lim KC (2003). Pattern of third molar impaction in a Singapore Chinese population: a retrospective radiographic survey.. Int J Oral Maxillofac Surg..

[6] Pell GJ (1933). Impacted mandibular third molars, classification and modified technique for removal.. Dental Digest..

[7] Ayranci F, Omezli MM, Sivrikaya EC, Rastgeldi Z (2017). Prevalence of third molar impacted teeth: a cross-sectional study evaluating radiographs of adolescents.. Journal of Clinical and Experimental Investigation..

[8] Hashemipour MA, Tahmasbi-Arashlow M, Fahimi-Hanzaei F (2013). Incidence of impacted mandibular and maxillary third molars: a radiographic study in a Southeast Iran population.. Med Oral Patol Oral Cir Bucal..

[9] KalaiSelvan S, Ganesh SKN, Natesh P, Moorthy MS, Niazi TM, Babu SS (2020). Prevalence and pattern of impacted mandibular third molar: an institution-based retrospective study.. J Pharm Bioallied Sci..

[10] Upadhyaya C, Chaurasia NK, Neupane I, Srivastava S (2017). Incidence and pattern of impaction of mandibular third molars: a single institutional experience in Nepal.. Kathmandu University Medical Journal (KUMJ)..

[11] Haque B, Bhandari K, Karna D (2021). Pattern of mandibular third molar impaction in different age groups and indications for removal..

[12] Bataineh AB, Albashaireh ZS, Hazza'a AM (2002). The surgical removal of mandibular third molars: a study in decision making.. Quintessence international (Berlin, Germany : 1985)..

[13] Tenrilili A, Yunus B, Rahman F (2023). Third molar impaction prevalence and pattern: a panoramic radiography investigation.. Jurnal Radiologi Dentomaksilofasial Indonesia (JRDI)..

[14] Thomas F, Renaud F, Benefice E, de Meeus T, Guegan JF (2001). International variability of ages at menarche and menopause: patterns and main determinants.. Hum Biol..

[15] Venta I, Turtola L, Ylipaavalniemi P (1999). Change in clinical status of third molars in adults during 12 years of observation.. J Oral Maxillofac Surg..

[16] Syed KB, Alshahrani FS, Alabsi WS, Alqahtani ZA, Hameed MS, Mustafa AB (2017). Prevalence of distal caries in mandibular second molar due to impacted third molar.. J Clin Diagn Res..

[17] Ishwarkumar S, Pillay P, Haffajee M, Satyapal K (2019). Prevalence of impacted third molars in the South African Indian population of the eThekwini Metropolitan Region.. South African Dental Journal..

[18] McArdle LW, Renton TF (2014). Distal cervical caries in the mandibular second molar: an indication for the prophylactic removal of the third molar?. Br J Oral Maxillofac Surg..

[19] Garaas RN, Fisher EL, Wilson GH, Phillips C, Shugars DA, Blakey GH (2012). Prevalence of third molars with caries experience or periodontal pathology in young adults.. J Oral Maxillofac Surg..

[20] National Institute for Health and Care Excellence. (2000). Guidance on the extraction of wisdom teeth [Internet]..

